# Biotechnological Applications of Scyphomedusae

**DOI:** 10.3390/md17110604

**Published:** 2019-10-24

**Authors:** Louise Merquiol, Giovanna Romano, Adrianna Ianora, Isabella D’Ambra

**Affiliations:** 1 Integrative Marine Ecology Department, Stazione Zoologica Anton Dohrn, Villa Comunale, 80121 Napoli, Italy; louise.merquiol@szn.it; 2 Marine Biotechnology Department, Stazione Zoologica Anton Dohrn, Villa Comunale, 80121 Napoli, Italy; giovanna.romano@szn.it (G.R.); ianora@szn.it (A.I.)

**Keywords:** collagen, fatty acids, crude venom, bioactive compounds, nutraceuticals, cosmeceuticals, biomedicals, biomaterials

## Abstract

As people across the world live longer, chronic illness and diminished well-being are becoming major global public health challenges. Marine biotechnology may help overcome some of these challenges by developing new products and know-how derived from marine organisms. While some products from marine organisms such as microalgae, sponges, and fish have already found biotechnological applications, jellyfish have received little attention as a potential source of bioactive compounds. Nevertheless, recent studies have highlighted that scyphomedusae (Cnidaria, Scyphozoa) synthesise at least three main categories of compounds that may find biotechnological applications: collagen, fatty acids and components of crude venom. We review what is known about these compounds in scyphomedusae and their current biotechnological applications, which falls mainly into four categories of products: nutraceuticals, cosmeceuticals, biomedicals, and biomaterials. By defining the state of the art of biotechnological applications in scyphomedusae, we intend to promote the use of these bioactive compounds to increase the health and well-being of future societies.

## 1. Introduction

By definition, biotechnology is ‘the application of biological knowledge and techniques to develop products and other benefits for humans’ [1]. Currently, biotechnology includes production processes and techniques as well as cutting-edge molecular and genomic biological applications. Since terrestrial habitats are generally overexploited, research in the last two decades has focused on marine organisms as they have been found to possess or synthesise bioactive compounds that often have no terrestrial counterparts and which may, therefore, find new biotechnological applications in different fields. 

**Figure 1:**
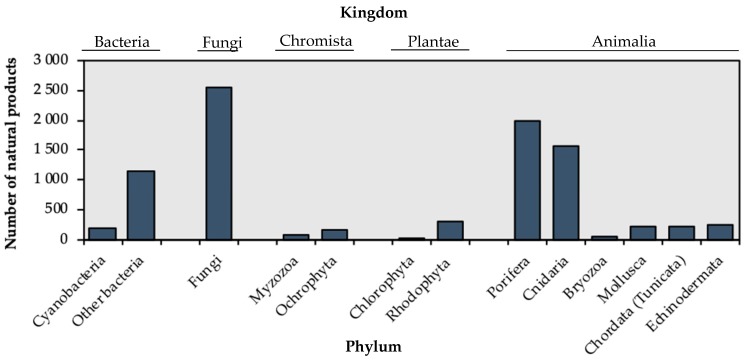
New natural products extracted from marine organisms between 2011 and 2017.

The ocean is an almost unexploited source of biological and chemical diversity. It covers more than 70% of the Earth’s surface and is inhabited by more than 194,000 described species of bacteria, plants and animals [2]. However, only a relatively small number of marine organisms have been exploited until now, providing about 9000 novel natural products between 2011 and 2017 [3,4,5,6,7,8,9,10,11] (Figure 1).

Cnidaria have been relatively under-exploited to obtain natural products compared to other taxa (Figure 1). Most of the natural products extracted from Cnidaria derive from benthic cnidarians, while a limited number of bioactive compounds have been extracted from pelagic cnidarians (hydromedusae and scyphomedusae) [3,4,5,6,7,8,9,10,11]. Nevertheless, the natural products synthesised by pelagic cnidarians may find several important applications for humans. 

The green fluorescent protein (GFP) is likely the most famous jellyfish-derived compound that is finding several applications in the biomedical field. The hydromedusa *Aequorea victoria* (Murbach and Shearer 1902), which was described in the waters of Victoria (Canada), appears colourless and transparent; nevertheless, it produces bright green flashes along the edge of its bell using GFPs, which extend the wavelength of the emitted light to the green spectrum. The ecological and evolutionary significance of the processes leading to light production in the ocean are still debated and little known. However, after their first extraction in 1962 [12], GFPs have found large applications in oncology and nerve cell development due to their ability to tag cells. The importance of this discovery and its applications led O. Shimomura, M. Chalfie and R. Tsien to the award of the Nobel Prize in Chemistry in 2008. 

In addition to GFPs, O. Shimomura isolated another calcium-activated photoprotein in *A. victoria*, aequorin, which, like GFPs, is responsible for light emission by this hydromedusa. However, while GFPs emit light in the green spectrum, emissions mediated by aequorin are in the blue wavelength. A sub-unit of aequorin, apoaequorin, is the main component of an integrator used to enhance neuronal activity and memory (www.prevagen.com).

Jellyfish (hydromedusae, scyphomedusae and ctenophores) have been considered as a nuisance or a “pest” for their interference with human activities at sea (tourism, fishery, farming, industries) and a dead end in marine food webs for a long time, but their role in marine ecosystems has been recently re-evaluated [13]. In recent decades, the abundance of jellyfish appears to have increased at a global scale [14,15,16]. The general increase and its potential causes are still under discussion due to the lack of regular monitoring of jellyfish abundance [17]. Several human-driven activities have been indicated to favour the increase of jellyfish, such as overfishing [18], eutrophication of coastal areas [19], and climate change [20], but it is likely that a combination of these has acted in concomitance with local patterns [21]. Jellyfish are often an undesired by-catch resulting from regular fishing activities, because they damage and break the nets in which they are accidentally caught. This by-catch, however, could ensure an abundant and regular availability of these organisms in certain coastal marine areas that could be exploited for several biotechnological applications.

## 2. Organic Content of Scyphomedusae

Scyphomedusae have been found to possess mainly three categories of compounds that may find biotechnological applications: collagen, fatty acids and bioactive compounds extracted from the crude venom of their nematocysts. Scyphomedusae contain a large volume of water (>95%, [22]). The quantity of organic matter, therefore, is reduced compared to water and varies greatly across species [22]. Scyphomedusae may have different salt content depending on seawater salinity. At high salinities, the relative abundance of salt compared to the organic matter in their body may be higher compared to the same species at low salinity. As a consequence, the estimation of the organic content in scyphomedusae may be biased by salt content if salt is not removed [23]. Comparisons across species are made difficult by the fact that analyses of all main compounds have not been carried out on the same organisms and often only certain tissues have been considered. Additionally, different approaches have been used to determine organic matter content [23]. Some studies have relied on the determination of organic content based on the wet mass (WM) of scyphomedusae. Because the water content itself varies greatly across individuals, the determination of the organic matter in terms of dry mass (DM) has been preferred (Table 1).

Overall, proteins account for most of the organic matter in scyphomedusae, although determination of the three classes of compounds in the same organism and the determination of carbohydrates are very limited compared to proteins and lipids (Table 1). Despite different determinations as DM or WM, it appears that the rhizostomes (Cnidaria, Scyphozoa, Rhizostomeae) contain more proteins than other scyphomedusae (Table 1). 

## 3. Proteins

The proteins found in scyphomedusae are mainly organized in the collagen complex. Collagen is a structural macro-protein which accounts for 20%–30% of all proteins, particularly in mammals [48]. Basically, collagen structure is made by three polypeptide chains arranged in three helixes, with two chains being identical (α1) and the third (α2) being different from the other two in its primary structure and sequence. Each chain has about 1050 amino acids with the triplet Gly-X-Y, where X and Y can be any amino acid. Glycine plays a key role to allow the three α chains to pack into hexagonal structures which then form elongated fibrils [49].

The complex structural and hierarchical organization allows amino acids to form more than 20 types of collagen [50]. The different types of collagen are distributed into diverse tissues (Table 2) and play a key role as a basic component of a variety of tissues, especially those with a structural function, such as cartilage and bone (Table 2). 

Terrestrial mammals possess a high content of collagen and have been exploited for this purpose. Nevertheless, determinations of collagen content in marine organisms have highlighted that fish skin and bones and sponges contain a remarkable amount of collagen and are currently considered an alternative to bovine- and porcine-derived collagen [51] (Table 3). However, within marine organisms, the yield of collagen obtained from some scyphomedusae is greater than the collagen obtained from sponges using the same extraction method (acid solubilisation; Table 4).

At present, most collagen is derived from the skin and bones of bovines and pigs. However, some issues have been raised regarding the use of terrestrial mammalian-derived collagen. Firstly, bovine-derived collagen may induce health problems in humans due to transfer of the bovine spongiform encephalopathy (BSE). Secondly, religious beliefs and alimentary restrictions may prevent some populations from using bovine- and porcine-derived products [62]. Finally, bovines and pigs are bred in captivity, often in unhealthy conditions, which have already raised the concern of animalist movements. Jelly-derived collagen could therefore be an attractive alternative to other forms of collagen. For example, the collagen extracted from *Rhizostoma pulmo* (Macri 1776) was used to build implantable scaffolds for tissue engineering that were successfully implanted into a mouse model [63]. Several assays indicated that jelly-derived scaffolds have optimal adsorption and biocompatibility properties. These results suggest that collagen derived from scyphomedusae is as biocompatible as bovine collagen [63].

Addad et al. [64] tested the cytotoxicity and adhesion of collagen extracted from *Rhizostoma pulmo* and compared it with type I collagen of bovine origin. By defining the mechanisms beyond cell interactions, the authors determined that integrins and heparin-sulfate receptors of human cells could recognize jelly-derived collagen, so that cells could adhere to jelly-derived collagen as they did to mammalian collagen. Based on the biocompatibility between jelly-derived and human collagen, it is likely that collagen extracted from scyphomedusae may be more suitable for human use than bovine and porcine collagen.

Different extraction methodologies result in different yields of collagen, with pepsin solubilisation being the most effective to maximize extraction processes [32,65]. Despite differences due to extraction protocols, it appears that rhizostome scyphomedusae such as the edible medusa *Rhopilema esculentum* Kishinouye 1891 are likely to provide a greater yield of collagen as they possess a higher content of collagen than other organisms, including sponges (Table 4). 

To date, the detailed amino acid composition of only a few tropical and Mediterranean species has been determined (Table 5). The most abundant amino acid in scyphomedusae is glycine, which is the essential amino acid that binds with other amino acids to form the basic triplets of the collagen structure. Other essential amino acids include histidine, isoleucine, leucine, lysine, arginine, methionine, phenylalanine, threonine and valine which are all found in different quantities in different species, with Rhizostomeae being apparently more enriched in essential amino acids than Semaeostomeae. Relevant amounts of histidine are found only in *Cotylorhiza tuberculata* (Macri 1776) and *Rhizostoma pulmo*, while this essential amino acid is reduced or not detectable in other scyphomedusae (Table 5). Other important amino acids, such as leucine, arginine and isoleucine are overall remarkably abundant in all species (Table 5). Tryptophan is totally lacking within the essential amino acid composition of scyphomedusae.

## 4. Fatty Acids

Fatty acids (FAs), the basic units of lipids, are made up by long chains of an even number of carbon atoms linked by a single bond in saturated fatty acids (SFAs) and double in unsaturated fatty acids. The number and position of the double bond determines the sub-groups: mono-unsaturated fatty acids (MUFAs) and poly-unsaturated fatty acids (PUFAs). FAs build up storage units of energy for both plants and animals, but they are also part of membranes and cell structures. They can be biosynthesized by the organism or taken up through the diet. In the latter case, FAs, differently from other complex molecules, are not broken down, and remain unchanged or slightly modified during the digestion process. As reservoirs, they usually do not undergo transformation during regular cell metabolism. Therefore, they are conservative tracers which have been finding large application in ecological studies to elucidate trophic interactions among organisms and to determine the flow of organic matter from the base of the food web (phytoplankton) to higher trophic levels [71]. 

Omega-3 (ω-3) and omega-6 (ω-6) are essential PUFAs which play a key role in building and keeping the integrity of cell membranes. Omega-3 are involved in crucial processes such as growth, development, and tissue and cell homeostasis [72]. Additionally, they have been shown to promote hypo-triglyceridemic, anti-inflammatory, antihypertensive, anticancer, antioxidant, anti-depressive, anti-aging, and anti-arthritic beneficial effects for humans [73]. Excessive amounts of ω-6 PUFAs and a very high ω-6/ω-3 ratio are found in contemporary western diets and appear to promote genesis of many diseases, including cancer, and cardiovascular, inflammatory and autoimmune diseases [74]. Conversely, increased levels of ω-3 PUFAs (a low ω-6/ω-3 ratio) help reduce the onset of these diseases [74].

In scyphomedusae, PUFAs appear to be overall more abundant than SFAs and MUFAs, which appear less abundant, despite the variability of the three compounds in different species (Table 6). The genus *Chrysaora*, *Cyanea* and *Stomolophus* possess a higher content in PUFAs than other scyphomedusae. The species *Pelagia noctiluca* (Forsskål 1775) appears to contain a reduced amount of PUFAs compared to all other species. The content of PUFAs in scyphomedusae is indeed overall low when the wet mass of medusae is considered. Based on the data by Leone et al. [32], the amount of PUFAs was 0.23 g/kg medusa WM in *Aurelia* sp. 1, 1.44 g/kg medusa WM in *Cotylorhiza tuberculata* and 0.41 g/kg medusa WM in *Rhizostoma pulmo* (Table S1). These amounts appear much lower than 158–904 g/kg WM of krill (*Euphausia superba* Dana 1850) [75]. At a first-order-approximation, thousands of scyphomedusae are necessary to obtain the yield of PUFAs provided by a kilo of krill. However, the exploitation of krill to produce oil and integrators may alter the delicate balance of Arctic and Antarctic ecosystems, where krill play a key role within the food web that sustains ecologically relevant predators such as whales [76]. Considering that outbreaks of scyphomedusae may be made up of several hundreds of individuals [16,77] and scyphomedusae may be reared in captivity at an industrial scale, their availability in large numbers may be more cost effective than in other organisms, which may make them an alternative sustainable source of PUFAs.

The ω-3 FAs are overall more abundant than ω-6, which results in an ω-6/ω-3 ratio < 1 in most scyphomedusae (Table 6). Only *Chrysaora quinquecirrha* (Desor 1848) and *Cyanea nozaki* Kishinouye 1891 show a ratio > 1 whereas *Rhizostoma luteum* (Quoy and Gaimard 1827) has a ratio slightly > 2. Therefore, most scyphomedusae possess a ratio comparable with healthy foods such as krill and fish, with a threshold value below 1 which is considered an indicator of a healthy diet [74]. 

## 5. Bioactive Compounds from Crude Venom 

Cnidarians possess specialised structures (nematocysts) which discharge the venom used to prey upon other organisms (mainly plankton) and as a defence from potential predators. The venom synthesised by pelagic cnidarians (Hydrozoa, Scyphozoa, Cubozoa) consists mostly in proteins, with phospholipase A_2_ and metalloproteases being the most common components of crude venom across the classes (Table 7).

Phospholipase A_2_ enzymes are commonly found also in mammals and in arachnids, insects, and snake venom. Excessive amounts of phospholipase A_2_ after the bite of an insect, an arachnid, a snake or after contact with scyphomedusae, cause arachidonic acid to be released from phospholipid membranes thereby inducing local inflammation and pain. In normal mammalian brain cells, phospholipase A_2_ regulates the conversion of arachidonic acid into proinflammatory mediators. When the regulatory activity of phospholipase A_2_ does not work properly or amounts of this enzyme are lower than normal, excessive amounts of proinflammatory mediators induce oxidative stress and neuroinflammation which can lead to neurological diseases such as Alzheimer, epilepsy, multiple sclerosis and ischemia [78].

The definition of metalloproteinases includes a large variety of proteinase enzymes, which share the need of a metal atom to induce their catalytic activity. In most pelagic cnidarians these enzymes have not been defined in detail nor has their atomic weight been determined (Table 7). Similarly to metalloproteinases, several proteinases have not been defined in detail (Table 7). 

Likely due to the diverse components, the crude venom extracted from cnidarians has a wide range of effects on humans. Cubomedusae (Cnidaria, Cubozoa) venoms have severe effects, which span from severe envenomation with extensive dermonecrosis and oedema, to diffused neurotoxicity, motorial and respiratory problems, cardiovascular symptoms, hypotension and occasionally death [85,86]. 

Given the potential lethal effects on humans, the study of venoms from cubomedusae is more advanced than in other cnidarians, particularly in the tropical and subtropical Atlantic, Pacific and Australia, where cubomedusae are numerous (Table 7). Of the cubomedusae, the sea-wasp, *Chironex fleckeri* Southcott 1956, can induce lethal cardiotoxicity within a few minutes after its sting, and has caused several deaths along the Australian coastline during the last century [87]. 

Of the Hydrozoa, only a very limited number of species is known to be harmful for humans. *Physalia physalis* (Linnaeus 1758) has cardiotoxic and neurotoxic effects on humans which can be as lethal as those induced by cubomedusae.

Except for *Cotylorhiza tuberculata*, which has been found to be harmless for humans, the effects of crude venom from other scyphomedusae on humans vary greatly (Table 7). Local skin damage appears to be induced by *Chrysaora quinquecirrha* and *Pelagia noctiluca*. Blooms of *Pelagia noctiluca* in the 1980s and 1990s in the Mediterranean Sea fuelled the study of the crude venom in the species (Table 7; [88]). Cytotoxic and cytolytic activities appear to be common to several species of scyphomedusae, that differ from cubomedusae, whose crude venom seems to have a hemolytic and neurotoxic effect in most cases (Table 7).

In addition to phospholipase A_2_, toxic compounds such as saxitoxin, gonyautoxin-4, tetrodotoxin and brevetoxin-2 have been reported in *Phyllorhiza punctata* von Lendenfeld 1884 [89]. However, since these compounds are known to be synthesized by dinoflagellates [90], it is likely that these toxins were produced by dinoflagellate endosymbionts of the scyphomedusa rather than the scyphomedusa itself.

## 6. Biotechnological Applications of Scyphomedusae: State of the Art and Perspectives

### 6.1. Nutraceuticals

Scyphomedusae are a common ingredient in the eastern cooking tradition. Once they are dehydrated with aluminium salts, they are cut in pieces or strips and sold in markets. For exportation to international markets, they are usually packed under vacuum and become the main or side component of salads and soups. Scyphomedusae have been fished for internal use and export in Thailand, Indonesia, Malaysia, Philippines, Japan and China for several centuries [91]. In China, the scyphomedusa *Rhopilema esculentum* sustains a multi-million-dollar fishery [92] and industrial activities involved in processing scyphomedusae for edible purposes [93]. 

Western countries have shown some resilience to the introduction of scyphomedusae into their diet because the traditional method used in eastern countries to preserve jellyfish may induce side health issues to humans and an alternative processing method has not been developed [94]. Nevertheless, the consumption of *Rhizostoma pulmo* as food in the Black Sea has been suggested to meet the decrease in fish landings and the co-occurring increase in scyphomedusae abundance [95]. The collapse of fish stocks and increased demand from Asian markets have led the Americas to expand jellyfish fisheries by targeting rhizostome scyphomedusae [96].

Recently, Leone et al. [97] have proposed a simple protocol to process scyphomedusae to use them as human food in western countries. Three Mediterranean scyphomedusae, *Aurelia coerulea* von Lendenfeld 1884, *Cotylorhiza tuberculata* and *Rhizostoma pulmo*, have undergone a heating treatment comparable to a mild food processing to evaluate the effects on the biochemical composition and antioxidant activity. Results have indicated that the thermal treatment is suitable for processing *R. pulmo*, because heating at 100 °C stabilizes protein content and increases antioxidant activity more than in the other two Mediterranean scyphomedusae. This finding confirms the feasibility of processing scyphomedusae in western countries to use them as an alternative/integrative source of food.

Large consumption of scyphomedusae has been indicated as one of the reasons for the healthier lifestyle and better aging of eastern compared to western populations. Indeed, the analysis of scyphomedusae tissue has confirmed that they are rich in proteins (mainly collagen), have low lipid content, low *ω*6/*ω*3 ratio, and low caloric content. Therefore, integrating the human diet with direct consumption or compounds derived from scyphomedusae is likely to increase the quality of the diet [44,97]. Nevertheless, at present exploitation of scyphomedusae is based on analysis of the biochemical composition of medusae, while rigorous experimental work to prove the benefits of a diet based on or including scyphomedusae is still lacking.

### 6.2. Cosmeceuticals

Considering that human populations are aging longer and contemporary society has made appearing young a cult that spans across ages, social classes and activities, the need for new, effective anti-age products has increased. Scyphomedusae may provide a relevant contribution to the cosmeceutical industry for at least two properties. Firstly, the biocompatibility between human and jelly-derived collagen [63] is a preliminary requirement that facilitates the application of collagen extracted from scyphomedusae in this field. Secondly, some rhizostome scyphomedusae have shown antioxidant activities that may be highly valuable in the cosmeceutical industry. The edible scyphomedusa, *Rhopilema esculentum*, has shown antioxidant activity [141], but also the Mediterranean rhizostomes, *Rhizostoma luteum* [44], *Rhizostoma pulmo* [142], and *Cotylorhiza tuberculata* [32,97] have been found to have antioxidant properties. Although the application of scyphomedusae for cosmeceuticals has been suggested [32,97], at present it remains a potential biotechnological application that still needs to be developed. 

### 6.3. Biomedical Applications

#### 6.3.1. Collagen

Non-scientific journals have suggested that the consumption of scyphomedusae in eastern countries has enhanced the treatment and reduced the negative effects of arthritis, hypertension, back pain, ulcers, digestion, swelling, skin health and weight loss [91], but at present a rigorous scientific assessment of the positive effects of a jelly-diet on humans is limited. 

Some of the empirical observations mentioned above have found confirmation in scientific studies. Type II-like collagen of the scyphomedusa *Stomolophus meleagris* Agassiz 1862 delayed the onset and defeated collagen-induced arthritis in animal models [143]. Collagen hydrolysate of the scyphomedusa *Rhopilema esculentum* has proved to be an antioxidant, able to chelate Cu^2+^ ions and an inhibitor of tyrosinase activity [141,144]. In addition, collagen and collagen hydrolysate from the same scyphomedusa protected mice skin from damage induced by photoaging due to ultraviolet (UV) radiation [145].

Collagen extracted from the rhizostome *Nemopilema nomurai* Kishinouye 1922, which is commonly consumed as food in China and Japan, had immunostimulatory effects on cultured cells [146]. The mechanism beyond the immunostimulatory activity was highlighted by Nishimoto et al. [147], who found that collagen extracted from scyphomedusae stimulated both transcription and translation to enhance immunoglobulin and cytokine production and Morishige et al. [148] observed this effect on cells in vivo. 

Besides the aforementioned applications, bioactive compounds from scyphomedusae may be used as biomaterials for prosthesis, encapsulating polymers for drug delivery and medical device production. A polymeric matrix based on the collagen extracted from the scyphomedusa *Catostylus tagi* (Haeckel 1869) has allowed the development of a microparticulate protein delivery system [149]. This suggests that marine collagen derived from scyphomedusae may be used to produce microparticles for the controlled release of therapeutic proteins [149]. 

The biocompatibility between human and jelly-derived collagen could also find applications to stimulate tissue regeneration. Refibrillized collagen of the scyphomedusa *Rhopilema esculentum* was used to design porous scaffolds for potential application in cartilage regeneration [150]. Song et al. [151] engineered porous scaffolds by freeze-drying and chemical cross-linking of acid-solubilised collagen extracted from *Nemopilema nomurai*. Biocompatibility was determined as attachment of human fibroblasts and immune response after implantation of scaffolds in vivo and resulted to be higher than collagen extracted from other sources. 

Three-dimensional and highly porous scaffolds were built by Lee et al. [152] by combining collagen derived from the scyphomedusa *Nemopilema nomurai* and hyaluronic acid to bind with bioactive molecules for various biomimetic modifications. Scaffolds could be made functional with biotin by incorporating avidin. This mechanism may allow the incorporation of various bioactive molecules such as DNA, growth factors, drugs, and peptides into the three-dimensional porous scaffolds. Vascular grafts were engineered using tubular scaffolds of collagen extracted from *Nemopilema nomurai* and polylactic-co-glycolic acid fibrils by freeze-drying and electro-spinning the mixture. The hybrid scaffolds showed improved mechanical strength of the collagen scaffolds, which resulted in the enhancement of vascular endothelial cell development [153].

In addition to collagen, Nudelman et al. [154] used Q-mucin glycoproteins from *Rhopilema nomadica* Galil 1990 and *Nemopilema nomurai* made of nanometric fibers to produce scaffolds. The nanometric fibers allowed them to control the mechanical, morphological and chemical properties of the scaffolds, which showed a very high degree of biocompatibility and biodegradability. Assays of cell proliferation indicated that the scaffolds were suitable for cardiac cell growth. By including silver within the nanofibers, antibacterial activity was promoted, with formation of antibacterial mats. Pre-clinical trials on porcine wounds models resulted in complete healing of wounds.

An antimicrobial peptide was extracted from the mesoglea of *Aurelia aurita* Linnaeus 1758 [105]. The peptide, which was named Aurelin after the scyphomedusa, was shown to be active against Gram-positive and Gram-negative bacteria. The structure and activity of Aurelin suggest that it may find applications as antimicrobial and channel-blocking toxins [105].

#### 6.3.2. Crude Venom

Cnidarians are known since ancient times for their stinging activity. Despite the acute envenoming induced by some species, the extracts from crude venom may be used to develop antidotes. The venom of the most venomous jellyfish, *Chironex fleckeri*, contains bioactive proteins that trigger cell death [155]. By targeting hundreds of human genes, Lau et al. [155] recently identified the pathway of venom-exposed cell death and developed a novel venom antidote for *C. fleckeri* envenoming.

Although the immediate effects of scyphomedusae venoms are negative on human health (Table 7), a pioneering study in the mid-1970s highlighted that the crude venom extracted from scyphomedusae may contain bioactive compounds and suggested the potential for biomedical applications [156].

Extracts from crude venom of most scyphomedusae have cytotoxic and cytolytic activities (Table 7) (reviewed in [157]). These effects may be directed to damage targeted cells that cause problems to human health. Crude venom from *Nemopilema nomurai* had cytotoxic and cytolytic effects on heart and muscle myoblasts in mice and blood cells from different organisms, including humans [112]. More recently, an anti-tumoral activity was assessed in a model animal for extracts of crude venom from the same species [158]. *Cyanea nozakii* crude venom was toxic for colon cancer and hepatoma cells in humans [159]. 

Blooms of *Pelagia noctiluca* in the Mediterranean [88] have promoted several studies on the venom extracted from this species [160], which has shown cytotoxic and cytolytic effects on lung fibroblasts of Chinese hamsters, colon cancer in humans [161], glioblastoma in humans [162] and kidney cells [163]. Although the venom from *Rhizostoma pulmo* is considered slightly or non-harmful for humans [157], the crude venom extracted from this species was cytotoxic for lung fibroblasts of Chinese hamster [164]. These studies suggest that the venom extracted from scyphomedusae may find applications in the development of anti-cancer drugs.

In addition to its cytotoxic and cytolytic effects, crude venom extracted from *P. noctiluca* was found to have analgesic and antibutyrylcholinesterasic activity [165], and to induce oxidative stress on neuronal-like cells [166]. Bioactive compounds extracted from crude venom of *P. noctiluca* led to pore formation in targeted cell membranes, which resulted in osmotic lysis [167]. 

### 6.4. Biomaterials 

Collagen is the main component not only of living organisms, but also of inanimate objects. The potential of collagen derived from scyphomedusae in tissue regeneration and engineering has been discussed above (Section 6.3.1). Collagen extracted from jellyfish may become the main component of objects that have been overexploited, such as paper. Jelly-derived paper may integrate the recycling of wood-derived paper. To our knowledge, an industry in Israel tried to exploit scyphomedusae to produce paper-based products, but apparently the results did not fulfil the promising expectations. 

Cheng et al. [68] proposed using the collagen isolated from the mesoglea of the scyphomedusa *Rhopilema esculentum* to produce haemostatic materials to control massive blood loss. Collagen extracted from the scyphomedusa mesoglea resulted to be type I collagen that exhibited higher water absorption rates than medical gauzes. These results suggest that collagen extracted from scyphomedusae may become a haemostatic material for wound-healing applications. 

Very recently, Steinberger et al. [168] proposed using jellyfish biomass to produce bioplastics. According to the authors, “green” protocols can be used to extract collagen and Q-mucin glycoproteins from jellyfish. These compounds are suitable for the production of highly biodegradable plastics whose properties can be enhanced using cross-linkers derived from renewable resources. Given that the pollution due to plastics is a cogent issue of current societies, an environmentally friendly, sustainable production of highly biodegradable plastics may find a large variety of applications at present and in the future.

## 7. Conclusions

Modern society is in urgent need of finding alternative sources of bioactive compounds to replace overexploited resources. Marine organisms are providing an increasing number of natural products that may improve human health and well-being. Scyphomedusae have been considered a nuisance to human populations due to their interference with human activities along the coast. However, the positive effects of a diet that includes scyphomedusae as food are being supported by scientific studies that are confirming the nutraceutical value of scyphomedusae as well as their application in the cosmeceutical, biomedical and biomaterial fields. Despite the fact that some steps toward the exploitation of scyphomedusae have been made, the pipeline leading to their full exploitation is still in its infancy, especially in the biomedical field, which requires clinical trials on humans. Considering that scyphomedusae are abundant in marine coastal ecosystems, they may become a valuable resource for a variety of applications that may significantly improve the well-being of future populations.

## Figures and Tables

**Table 1:** Biochemical composition of scyphomedusae as percentage of dry mass (DM) or wet mass (WM) of different tissue types in scyphomedusae: B, bell; BM, bell margin; OA, oral arms; G, gonads; MG, male gonads; FG, female gonads; T, tentacles; W, whole body.

Species	Tissue	Proteins	Lipids	Carbohydrates	Proteins	Lipids	Carbohydrates	References
		(% Medusa WM)			(% Medusa DM)			
Semaeostomeae								
*Aurelia aurita*	W		0.5			0.03		[24]
	W		0.4					[25]
	W		0.2					[26]
	W	4.7	9.2	13.5	5.3	2	3.4	[27]
	W	5.9	1.9	2.9				[28]
	G	23.7		14.6				[28]
	OA	7.3		2.6				[28]
	B	4.2		1.5				[28]
	W	2.1–28.6	1.2–3.4	0.4–1.1				[29]
	G	4.4–23.0	2.6–6.0	1.1–2.1				[29]
	OA	4.1–15.3	1.3–4.0	0.6–1.5				[29]
	B	2.3–8.3	0.9–2.9	0.3–0.9				[29]
	W					0.03		[30]
	W		0.7			0.04		[30]
	W	3.5	0.4	19.9				[31]
*Aurelia* sp.1	W	5.7	4.1					[32]
*Chrysaora hysoscella*	W		2.7					[26]
*C. pacifica*	W	7.5	0.7	22.7				[31]
*C. quinquecirrha*	W					0.2		[24]
	MG					6.1		[24]
	FG					5.5		[24]
	T					4.1		[24]
*Cyanea capillata*	W	16.5	0.5	0.9				[33]
	G	28.4	0.6	0.9				[33]
	OA	29.8	1.2	1.1				[33]
	B	7.9	0.2	0.8				[33]
	G	9.6	1.6	1				[34]
	W		0.3–0.8					[35]
*C. lamarckii*	W		0.7					[26]
*Pelagia noctiluca*	W	10.9–19.8	1.3–2.9	0.1–0.7				[36,37]
	W					0.2		[38]
*Poralia rufescens*	W	0.2	0.4	0.1				[34]
*Stygiomedusa gigantea*	W		10.2			0.5		[39]
Rhizostomeae								
*Acromitus maculosus*	OA	33.7	1.1	6	1.3			[40]
	B	21.4	0.4	17.7	0.8			[40]
*Catostylus tagi*	W					0.4		[41]
	OA				4.3	0.5		[41]
	B				1.8	0.2		[41]
*Cotylorhiza tuberculata*	W	2.2	12.3					[32]
	G	36.8	6					[42]
	OA	20	6.4					[42]
	B	12	0.7					[42]
	BM	7.6	0.5					[42]
*Rhizostoma octopus*	W	12.8	0.3	0.8				[33]
	G	12.1	0.6	0.9				[33]
	OA	13.4	0.3	0.7				[33]
	B	6.6	0.3	0.7				[33]
*R. pulmo*	W		2.3					[43]
	W	6	4					[32]
	G	18	1.2					[42]
	OA	27	0.8					[42]
	B	8.7	0.7					[42]
	BM	13.7	1					[42]
*R. luteum*	W	0.8–1.9						[44]
*Rhopilema hispidum*	OA	43.8	1.4	10.7	2			[40]
	B	19.9	0.5	18.2	0.5			[40]
*R. esculentum*	OA	53.9	1.8	7.7	2.8			[40]
	B	38.1	0.6	8.9	1.6			[40]
*Stomolophus meleagris*	B				1.1			[45]
	M				1			[45]
Coronatae								
*Atolla wyvillei*	W		1.1					[46]
	W	16.9	4.2	1.7	0.8	0.2	0.1	[47]
	W		0.3		0.01			[39]

**Table 2:** Distribution of different types of collagen within tissues of mammals.

Collagen Type	Tissue
I	bone, dermis, tendon, ligaments, cornea
II	cartilage, vitreous body, nucleus pulposus
III	skin, vessel walls, reticular fibres of most tissues (lungs, liver, spleen)
IV	basement membranes
VI	cornea (often associated with type I collagen)

**Table 3:** Content of collagen types I, II and IV (percentages) in marine organisms determined by using two extraction methodologies, by solubilisation using either acid or pepsin.

Collagen Type	Species	Tissue	Collagen Content		References
			Pepsin	Acid	
I	*Priacanthus tayenus*	Bone		1.6	[52]
		Skin		10.9	[52]
	*Mystus macropterus*	Skin	28.0	16.8	[53]
	*Syngnathus schlegeli*	Skin	33.2	5.5	[54]
	*Lagocephalus gloveri*	Skin	54.3		[55]
	*Takifugu rubripes*	Skin	44.7	10.7	[56]
	*Saurida* spp.	Scales		0.79	[57]
	*Trachurus japonicus*	Scales		1.5	[57]
	*Mugil cephalis*	Scales		0.4	[57]
	*Cypselurus melanurus*	Scales		0.7	[57]
	*Dentex tumifrons*	Scales		0.9	[57]
	*Illex argentinus*	Skin		53	[58]
	*Sepiella inermis*	Skin	16.2	0.6	[59]
II	*Chiloscyllium punctatum*	Cartilage	9.6	1.3	[60]
	*Carcharhinus limbatus*	Cartilage	10.3	1.0	[60]
IV	Marine sponge			30	[51,61]

**Table 4:** Collagen content (percentage of dry (DM) or wet mass (WM)) in different species of scyphomedusae using two different extraction protocols based either on acid or pepsin solubilisation. B, bell; OA, oral arms; W, whole; M, mesoglea.

Species	Tissue	Collagen Content				References
		Pepsin		Acid		
		(% DM)	(% WM)	(% DM)	(% WM)	
*Aurelia aurita*	W		0.01			[64]
*Chrysaora* sp.	B		9–19			[65]
*Pelagia noctiluca*	W		0.07			[64]
*Catostylus tagi*	B	2.7				[66]
*Cotylorhiza tuberculata*	B		4.5			[64]
	OA		19.4			[64]
	B	<10				[64]
*Rhizostoma pulmo*	B		8.3–31.5			[64]
	OA		26–90			[64]
	B	<10				[64]
*Rhopilema asamushi*	-	35.2				[67]
*Rhopilema esculentum*	M		0.28		0.12	[68]
*Stomolophus meleagris*	M	46.4				[69]
*Nemopilema nomurai*	M	2.2				[70]

**Table 5:** Amino-acid (AA) composition of collagen extracted from scyphomedusae (residuals/1000 residues), * values in mg of AA per g of DM, ** values in mg of AA per g of protein. n.d., not detectable. B, bell; OA, oral arms; W, whole body.

	Semaeostomeae						Rhizostomeae								
Species	*Aurelia* sp.	*Aurelia aurita*	*Chrysaora* sp.	*Chrysaora hysoscella*	*Chrysaora Pacifica*	*Pelagia noctiluca*	*Catostylus Tagi*		*Cotylorhiza Tuberculata*		*Rhizostoma Pulmo*		*Rhopilema esculentum*	*Stomolophus Meleagris*	*Nemopilema Nomurai*
				*				**	**			*			
Tissue	W	W	W	W	W	W	W	B	OA	W	W	W	W	W	W
Amino-acids															
Hydroxyproline	-	-	70	-	-	-	65	21.9	16.9	-	-	-	-	40	57
Aspartic acid	20	94	76	12.2	86	6.9	84	97.5	98.4	25	32	8.4	68	79	71
Serine	60	46	44	6.2	46	2.9	42	48.2	50.3	55	67	3.9	44	45	45
Glutamic acid	87	138	101	17.6	139	10.3	115	141.3	152.2	160	152	12.9	86	98	94
Glycine	352	145	320	19.6	166	13.5	269	94.2	89.3	59	53	8.4	268	309	344
Histidine	n.d.	12	n.d.	2.5	14	0.9	-	8.8	12	78	56	1.4	6	2	1
Arginine	7	69	58	8.3	64	5	62	77.7	68.7	-	20	6.4	77	52	57
Threonine	64	50	34	6	45	3.1	31	48.2	46.3	74	50	4.3	36	35	28
Alanine	45	67	87	6.5	66	4.1	101	70.1	64.7	43	39	4.7	109	82	77
Proline	27	104	79	6.2	107	4.1	78	75.6	68.1	51	39	5.1	72	82	79
Cystine	26	5	n.d.	-	4	-	1	12	10.9	-	13	-	3	-	-
Tyrosine	60	29	10	4.6	30	1.8	4	28.5	31.5	70	76	2.6	18	6	5
Valine	43	36	22	6	36	3.1	24	44.9	46.3	59	49	4.3	38	35	24
Methionine	38	15	16	-	19	-	5	18.6	19.5	53	46	-	12	4	8
Lysine	60	68	17	10.4	64	4.9	29	72.3	76.7	61	69	7	51	38	24
Isoleucine	43	32	23	5.5	33	2.6	22	36.1	37.2	57	55	3.5	31	22	16
Leucine	n.d.	44	31	7.8	56	3.6	31	56.9	62.4	74	91	5.1	42	34	27
Phenylalanine	66	44	14	5.3	25	2.1	6	36.1	42.3	80	93	3.3	30	10	8
Hydroxylysine	-	-	-	-	-	-	32	11.2	6.3	-	-	-	-	27	35
Tryptophan	n.d.	-	-	-	-	-	-	-	-	n.d.	n.d.	-	0	0	0
Reference	[32]	[31]	[65]	[23]	[31]	[23]	[66]	[66]	[66]	[32]	[32]	[23]	[68]	[69]	[70]

**Table 6:** Content of the main groups of fatty acids (SFAs, saturated fatty acids; MUFAs, mono-unsaturated fatty acids; PUFAs, poly-unsaturated fatty acids) as percentage of total fatty acids in scyphomedusae. B, bell; OA, oral arms; W, whole body.

Species	Tissue	Total SFAs	Total MUFAs	Total PUFAs	ω-3	ω-6	ω-6/ω-3	Location	References
Semaeostomeae									
*Aurelia aurita*	W	29.4	37.1	27.9	18	10	0.6	NW Atlantic	[79]
	W	54	13.8	32.3	26.3	5.8	0.2	Irish Sea	[80]
	W	46.7	19.2	28	20.9	7.1	0.4	Seto Inland Sea	[81]
	W	29.8.1	12	57.2	38.5	18.7	0.5	Yellow Sea	[82]
	W	41	8.4	33.4	11.3	16.9	0.7	New Zealand	[83]
	W	53.4.3	12.2	30.7				Tokyo Bay	[31]
	W	76.7	15.3	3.9				Ionian Sea	[30]
*Aurelia* sp.1	W	69.5	4.7	25.8	19	6.8	0.4	NW Mediterranean	[32]
*Chrysaora hysoscella*	W	22.7	22.4	55	47.1	6.2	0.1	Irish Sea	[26]
*Chrysaora pacifica*	W	45.9	13	35.3				Tokyo Bay	[31]
*Chrysaora quinquecirrha*	W	23.5	8.2	59.5	23.6	35.9	1.5	Charleston harbour	[24]
*Cyanea lamarckii*	W	40.2	19.2	40.8	30.1	9.1	0.3	Irish Sea	[26]
*Cyanea capillata*	W	26.1	23.3	47.4	34.6	12.7	0.4	NW Atlantic	[35]
*Cyanea nozakii*	W	29.9	6	57.9	26.9	30.5	1.1	Yellow Sea	[82]
*Pelagia noctiluca (medusae)*	W	63.4	21.1	10.2	4.8	3.8	0.8	NW Mediterranean	[84]
*Pelagia noctiluca (ephyrae)*	W	33	11	52.1	40.6	10.7	0.3	NW Mediterranean	[84]
*Stygiomedusa gigantea*	W	24.2	41.3	31	28.5	2.5	0.1	Antarctic	[39]
Rhizostomeae									
*Cotylorhiza tuberculata*	W	54.8	15.2	30	16.4	13.6	0.8	NW Mediterranean	[32]
*Rhizostoma luteum*	W	30.2	20.8	49	15.6	33.4	2.1	NW Mediterranean	[44]
*Rhizostoma octopus*	W	59.8	15.3	25.1	20.7	4.4	0.2	Irish Sea	[26]
*Rhizostoma pulmo*	W	68.2	7	24.8	13.5	11.3	0.8	NW Mediterranean	[32]
*Stomolophus meleagris*	W	23	6.8	59.9	39.7	20.2	0.5	Charleston harbour	[24]
	B	36.8	6.4	56.8	38.2	18.4	0.5	Yellow Sea	[82]
	OA	35.6	4.5	59.9	38.1	21.3	0.6	Yellow Sea	[82]
Coronatae									
*Atolla wyvillei*	W	30.9	30.6	34.2	31.1	3.1	0.2	Antarctic	[39]

**Table 7:** Main components and molecular mass (kDa) of crude venom extracted from different species of Scyphozoa, Cubozoa and Hydrozoa and their biological activity.

Species	Venom Main Component	Molecular Mass (kDa)	Biological Activity	References
Scyphozoa				
*Aurelia aurita*	Phospholipase A_2_		Cytolytic Hemolytic, neurotoxic, myotoxic, local skin irritation	[98,99]
	Proteolytic enzymes			[100]
	Tetramine and unidentified protein		Dermotoxic, temporary paralysis, oedema	[101,102]
	TX-1	54		[103]
	TX-2	51		[103]
	Metalloproteinases		Gelatinolytic, caseinolytic, fibrinolytic	[104]
	Aurelin	4.30		[105]
*Cassiopea andromeda*	Phospholipase A_2_		Hemolytic, dermonecrotic, local skin irritation	[98]
*C. xamancha*	Phospholipase A_2_		Hemolytic, dermonecrotic, local skin irritation	[98]
*Cotylorhiza tuberculata*			Unharmful	[106]
*Chrysaora hysoscella*	Cationic protein		Dermotoxic, cytotoxic	[107]
*C. quinquecirrha*	DNase	110	Dermonecrotic, cytotoxic	[108]
	Acid protease	120–150		[108]
	Alkaline protease (metallopeptidase)	100		[108]
	Collagenase			[108]
*Cyanea capillata*	Basic protein(s)	70	Cardiotoxic, dermonecrotic, musculotoxic	[108,109]
	CcTX-1	31.173	Cytotoxic	[110]
	CcNT	8.22	Neurotoxic	[110]
	Phospholipase A_2_		Cytolytic, cytotoxic, hemolytic	[99,111]
*C. lamarckii*	ClGP-1	27	Cytotoxic	[110]
	Phospholipase A_2_		Cytolytic, cytotoxic, hemolytic	[111]
*C. nozakii*	Metalloproteinases		Gelatinolytic, caseinolytic, fibrinolytic	[104]
*Nemopilema nomurai*	Metalloproteinases	28–36	Gelatinolytic, caseinolytic, fibrinolytic	[104]
		20–40/10–15	Cytotoxic, hemolytic	[112]
*Pelagia noctiluca*	Proteinaceous macromolecules	44–66	Hemolytic, cytotoxic, dermonecrotic, hemolytic, local tissue damage	[113,114,115,116,117]
*Phyllorhiza punctata*	Phospholipase A_2_ Saxitoxin * Gonyautoxin-4 * Tetrodotoxin * Brevetoxin-2 *		Neurotoxic	[89]
*Rhizostoma pulmo*	Rhizoprotease	95	Proteolytic, hemolytic	[118]
	Rhizolysin	260	Hemolytic	[119]
			Cytotoxic, hemolytic	[120]
*Rhopilema esculentum*	Metalloproteinases		Gelatinolytic, caseinolytic, fibrinolytic	[104]
	Hyaluronidase	55–95	Degradation of extracellular matrix components	[104]
			Proteolytic, cytotoxic, hemolytic	[121,122]
*R. nomadica*	Phospholipase A_2_		Hemolytic	[123]
	Serine protease		Local skin damage	[124]
*Rhopilema* sp.	Phospholipase A_2_		Hemolytic	[110]
*Stomolophus meleagris*	SmP90	90	Radical scavenging	[110]
	Phospholipase A_2_, C-lectin, ShK, K_v_^+^ toxin, Metalloproteinases		Cytotoxic, cytolytic, hemolytic, local tissue damage	[125]
Cubozoa				
*Alatina moseri*	CaTX-A	43	Hemolytic	[103]
*Carybdea alata*	CaTX-A (CAH1)	43	Hemolytic Hemolytic	[110,126,127]
	CaTX-B	45		
*C. marsupialis*	Haemolysin	102–107	Cytolytic Hemolytic Hemolytic	[110,128,129]
	CmHl5	220		
	CmHl1	139		
	CmHl7	36		
	CmNt	120	Neurotoxic, hemolytic	[129]
*C. rastonii*	Phospholipase A_2_		Cytolytic Hemolytic Hemolytic	[99]
	CrTX-II			[130]
	CrTX-III			[130]
	CrTX-A	43		[103,110,126]
	CrTX-B	46		[110,126]
*Carukia barnesi*	Phospholipase A_2_		Cytolytic, hemolytic	[99]
	CbTX-I	21.67	Neurotoxic	[131]
	CbTX-II	18.16	Neurotoxic	[131]
*Chironex fleckeri*	Phospholipase A_2_		Cytolytic, hemolytic	[99]
	Metalloproteinases	17–130		[132]
	CfTX-1	43	Cardiotoxic, cytotoxic, dermonecrotic, lethal	[103,110,132,133,134]
	CfTX-2	45		
	CfTX-A	40		
	CfTX-B	42		
	CfTX-Bt	31.293		[103]
*Chiropsalmus quadrigatus*	CqTX-A	44	Hemolytic, neurotoxic, myotoxic	[110,135,136]
*Malo kingi*	MkTX-A	48.55	Dermonecrotic, inflammatory	[131]
	MkTX-B			
Hydrozoa				
*Hydra magnipapillata*	CqTX-A			[103]
*H. viridissima*	Hydralysin	27	Neurotoxic, cytolytic, paralytic	[137]
*Millepora* sp.	Phospholipase A_2_		Cytolytic, hemolytic	[99]
*Obelia geniculata*	Phospholipase A_2_		Cytolytic, hemolytic	[99]
*Olindias sambaquiensis*	Oshem1	3.013	Hemolytic Hemolytic	[110]
	Oshem2	3.376		[110]
	Metalloproteinases		Cytolytic, neurotoxic	[138]
*Physalia physalis*	Phospholipase A_2_			[139]
	Phospholipase B			[139]
	Physalitoxin	220	Hemolytic	[110,140]
	P1	220	Neurotoxic Neurotoxic	[110]
	P3	85		[110]
	PpV9.4	0.55	Hemolytic Neurotoxic, cardiotoxic	[110]
	PpV19.3	4.72		[110]
	Elastase Histamine		Musculotoxic, cytolytic, hemolytic	[108]
	Collagenase	25	Cytolytic, hemolytic	[108]
	DNase	75		[108]
*Tubularia larynx*	Phospholipase A_2_		Cytolytic, hemolytic	[99]

* Compounds likely synthesised by endosymbionts of the scyphomedusa.

## Supplementary Materials

The following are available online at https://www.mdpi.com/1660-3397/17/11/604/s1: Table S1: Calculations of total lipids and polyuns aturated fatty acids (PUFAs) content in three Mediterranean scyphomedusae.
