# Supplementary material for: Biotechnological Applications of Scyphomedusae
Source: Mar Drugs. 2019 Oct 24;17(11):604. doi: 10.3390/md17110604 (PMC6891278; doi:10.3390/md17110604)
Supplement: Supplementary file 1 [file marinedrugs-17-00604-s001.pdf]

Table S1. Calculation of total lipids and polyunsaturated fatty acids (PUFAs) content in three Mediterranean scyphomedusae.

Scyphozoan Species	Average Wet Mass Per Medusa (g) *	Dry Mass (% WM) *	Total Lipids		PUFA		
			(g/kg DM) *	(g/kg WM)	(% of total lipids) *	(g/kg WM)	(g/medusa WM)
Aurelia sp.1	257	2.2	41	0.90	25.8	0.23	0.06
Cotylorhiza tuberculata	638	3.9	123	4.80	30.0	1.44	0.92
Rhizostoma pulmo	860	4.1	40	1.64	24.8	0.41	0.35

* data from Leone et al. [32]
